# Some Practical Suggestions about Plaster of Paris

**Published:** 1891-03

**Authors:** Rodrigues Ottolengui


ARTICLE VI.

### SOME PRACTICAL SUGGESTIONS ABOUT PLASTER OF PARIS.

BY RODRIGUES OTTOLENGUI.

The columns of our journals are more often given up to operative dentistry, or to theoretical discussions of supposed to be scientific matters, than to the every-day needs of the worker at the laboratory bench. We visit our neighbor, and he takes us cheerfully into his office, but infrequently invites us into his workshop—perhaps because it is dirty. Thus by association we learn more about each other's methods at the chair than we do of what we once called mechanical dentistry, which nowadays is too often relegated to inexperienced students or assistants. Whatever I do for my patients, I do with my own hands. Every dentist should be able to say the same, or at least to do the same. If anything presents which my hands could not accomplish, I do not hesitate to recommend my patient to the proper specialist, rather than to hire said specialist to come in and do the work surreptitiously, taking the credit myself. But this is not practical talk, which my title promises.

In Philadelphia once, I heard Prof. Essig say that he doubted if there were more than five men in Philadelphia who knew how to mix plaster. A sweeping assertion, which was however probably correct. Though at the moment in that city, I could not at that time have contended for a place among the learned five. What I know on the subject now is due to my association with Dr. Kingsley, who perhaps has no superior in the manipulation of this material.

First, then, for a few facts: Plaster will set more rapidly if tepid (not hot) water is used, than if we take cold. A clean smooth cup is the most convenient vehicle in which to mix plaster, rubber bowls being especially unsatisfactory to one who has become accustomed to the thin cup. New cups should be obtained as often as the surfaces become checked so that the plaster is difficult to remove. If an excess of material has been used, scrape it out into the waste box, and what little there is left in the cup will easily come away when hard, by filling the cup with water.

To mix plaster, put in the cup at the outset about as much water as judgment indicates will be needed. To add water later is a mistake. Drop in the plaster a little at a time, allowing it to disappear below the surface before adding any more; repeat this till the plaster protrudes above the water, which refuses to take up any more. Then stir vigorously with a smooth *clean* knife and a mixture moderately stiff, and perfectly smooth will reward you. The more it is stirred the more quickly it will set, and the stiffer it gets. Use it for the purpose in hand when it has reached the required consistency. If on mixing it is found that a miscalculation has been made as the mass is thin, do not add more plaster; such a step will ruin everything. Pour off any excess of water, and then stir till stiffening begins. Mix enough plaster at the outset, because to mix a second batch, especially in flasking, gives a mass one part of which will set sooner than the other, which is often very undesirable. In taking an impression of a large surface, this sometimes becomes necessary. Then mix the first batch quite thin and flow it over the entire surface of the object of which a duplicate is desired. Then add other plaster until the impression is as thick as needed. By the time the last lot has set, the first will usually be hard, and all will be well.

In mixing for impressions of the mouth, use fine plaster, add a little salt and a little powdered pigment; Venetian red or Spanish brown serve admirably. This will produce a thick setting tinted impression material, which can be readily detected from the model which is to be poured into it. The plaster must be mixed as described, care being taken not to have it too thick. Do not stir until in the presence of the patient.

Then stir until it can be felt that the mass has begun to stiffen. Fill the cup rapidly, carry it to place firmly and hold it there motionless. If there is a high roof put some plaster in the valut with the knife. If it is feared that the buccal portions of the molar teeth may not be reached, place plaster there also, before inserting the tray. As soon as the plaster begins to set in the cup, chop it up so that it may be more readily removed when the cup is to be cleaned. As soon as the material in the cup will fracture sharply, remove the impression regardless of possible fracturing. If this does occur, collect all the pieces from the mouth; they may be replaced so as to form a practically perfect model. I have made up an impression from thirty odd pieces and secured good adaptation.

To procure a lower impression, put but little plaster in the tray, place plaster around the teeth in the mouth with the knife, being sure to cover all surfaces of the teeth; then quickly insert the tray and let the mass harden, and remove as before. In very difficult cases, where the teeth are long and perhaps loose, dispense with the tray altogether. When the material is sufficiently set, split it into sections with a sharp pen-knife, and afterwards join the fractured pieces together as before.

To pour a model, use a shaving brush, and with soap-lather, thoroughly soap the surface of the impression, especially in the pits for the teeth, and be careful to wash all the suds off afterward; otherwise the surface of the model will be pitted. Mix the plaster moderately thin. Have the surface of the impression saturated, and a little water in the pits for the teeth. Start the plaster into the last molar and let it fill up and run over into the other pits, forcing out the water ahead of it until all are filled. Then turn the impression over and shake the plaster out of the impression back into the cup. This insures a thin film of plaster over the entire surface. If the process is now repeated and the impression slowly built up without turning it over, the result will be a most accurate model.

Where the model is to be used for gold work, and dies must be made, if there are any teeth present, a pin should be placed in each. In doing this cut the heads off, and later the plaster teeth may all be broken off, the pins withdrawn, the dies made, and the teeth readily returned to position, where they may be again fastened by using a camel's hair brush and plaster very thin. In repairing models in this way, take a saucer with a little water and drop in a little plaster, but do not stir it. The plaster may be taken up with the brush placed along the joint between tooth and model, and as it soaks in the excess must be wiped away with the brush filled with water.

When it becomes desirable to trim a model, after it has lain around a day or two, and so become quite hard, dip it in water for a few minutes, and it may be readily cut with a sharp pen-knife. If an impression is filled and allowed to stand over night, drop it into hot water, and the steam generated will make separation more easy. Models should always be well soaked before more plaster is added to them. If, however, it is desired to preserve a model, upon which a plate is to be vulcanized, it may be readily done by soaping it slightly and flasking it as dry as possible. With care, after vulcanization, the investment may be removed without injury to the model, which, if then allowed to dry, may be separated from the plate.

To mix plaster and sand for investment of gold pieces, drop sand into water, and without stirring pour off as much water as will run out. If now half the quantity of plaster be added, vigorous stirring will produce a mass of the proper consistency. At first it will appear stiff, but by stirring the sand will soon yield enough water to saturate the plaster, when the whole will become incorporated.

Never varnish plaster under any circumstances, except for moulding in sand.—*Dental Mirror.*

